# 2-(4-Amino­phen­yl)-1,3-benzothia­zole

**DOI:** 10.1107/S1600536808031565

**Published:** 2008-10-04

**Authors:** Yong Zhang, Zhen-Hong Su, Qing-Zhi Wang, Lei Teng

**Affiliations:** a School of Chemical and Materials Engineering, Huangshi Institute of Technology, Huangshi 435003, People’s Republic of China; b Medical School, Huangshi Institute of Technology, Huangshi 435003, People’s Republic of China

## Abstract

The title compound, C_13_H_10_N_2_S, contains two independent mol­ecules in its asymmetric unit, with slightly different conformations. In one mol­ecule, the dihedral angle between the benzothia­zole unit and the benzene ring is 6.73 (1)°, while the corresponding angle in the other mol­ecule is 1.8 (1)°. In the crystal structure, the mol­ecules are linked into layers by N—H⋯N hydrogen bonds.

## Related literature

For background concerning the medical applications of benzothia­zole compounds, see: Alfred & Sawhney (1968 ▶); Hutchinson & Jennings (2002 ▶).
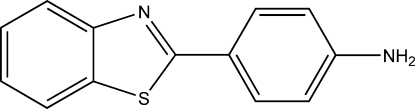


## Experimental

### 

#### Crystal data

C_13_H_10_N_2_S
*M* *_r_* = 226.29
Triclinic, 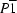

*a* = 8.7038 (5) Å
*b* = 9.5933 (6) Å
*c* = 14.5144 (9) Å
α = 70.720 (1)°
β = 77.326 (1)°
γ = 73.170 (1)°
*V* = 1084.63 (11) Å^3^
*Z* = 4
Mo *K*α radiation
μ = 0.27 mm^−1^
*T* = 298 (2) K
0.30 × 0.20 × 0.20 mm

#### Data collection

Bruker SMART CCD diffractometer
Absorption correction: multi-scan (*SADABS*; Sheldrick, 2003 ▶) *T*_min_ = 0.924, *T*_max_ = 0.948
6984 measured reflections
4199 independent reflections
3123 reflections with *I* > 2σ(*I*)
*R*_int_ = 0.036

#### Refinement

*R*[*F* ^2^ > 2σ(*F* ^2^)] = 0.046
*wR*(*F* ^2^) = 0.115
*S* = 0.97
4199 reflections
301 parameters
4 restraints
H atoms treated by a mixture of independent and constrained refinement
Δρ_max_ = 0.29 e Å^−3^
Δρ_min_ = −0.23 e Å^−3^

### 

Data collection: *SMART* (Bruker, 2001 ▶); cell refinement: *SAINT-Plus* (Bruker, 2001 ▶); data reduction: *SAINT-Plus*; program(s) used to solve structure: *SHELXS97* (Sheldrick, 2008 ▶); program(s) used to refine structure: *SHELXL97* (Sheldrick, 2008 ▶); molecular graphics: *SHELXTL* (Sheldrick, 2008 ▶); software used to prepare material for publication: *SHELXTL*.

## Supplementary Material

Crystal structure: contains datablocks global, I. DOI: 10.1107/S1600536808031565/bi2301sup1.cif

Structure factors: contains datablocks I. DOI: 10.1107/S1600536808031565/bi2301Isup2.hkl

Additional supplementary materials:  crystallographic information; 3D view; checkCIF report

## Figures and Tables

**Table 1:** Hydrogen-bond geometry (Å, °)

*D*—H⋯*A*	*D*—H	H⋯*A*	*D*⋯*A*	*D*—H⋯*A*
N1—H1*B*⋯N4^i^	0.819 (15)	2.644 (18)	3.374 (3)	149 (2)
N1—H1*A*⋯N3^ii^	0.850 (15)	2.324 (16)	3.145 (3)	163 (2)

Symmetry codes: (i) 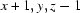; (ii) 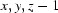.

**Fig. 1.**
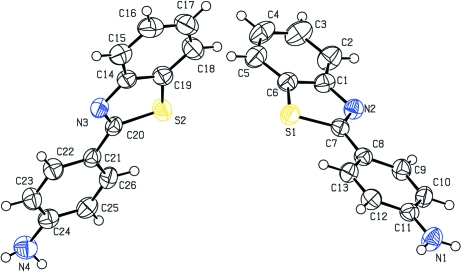
Molecular structure of the two independent molecules in the title compound. Displacement ellipsoids are drawn at the 50% probability level for non-H atoms.

**Fig. 2.**
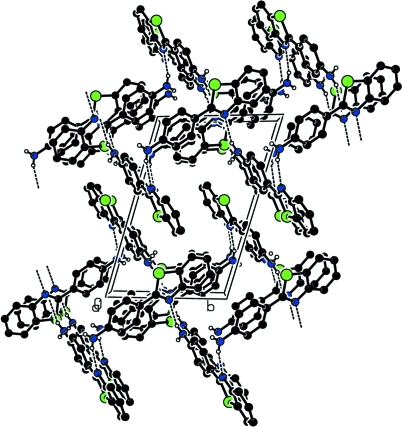
Packing diagram showing molecules linked by N—H···N hydrogen bonds (dashed lines) into layers in the (001) planes.

## supplementary crystallographic information

### Comment

Benzothiazole derivatives that contain five-membered sulfur-containing heterocyclic rings have been drawing great attention due to their antimalarial and antitumor properties (Alfred & Sawhney, 1968; Hutchinson & Jennings, 2002). Our research group is trying to prepare some benzothiazoles to find new antitumor compounds. In this context, we have crystallized the title compound and report its crystal structure.

### Experimental

All reagents and solvents were used as obtained without further purification. 4-Aminobenzoic acid (13.7 g, 0.1 mol) and 2-aminothiophenol (12.5 g, 0.1 mmol) were mixed together with polyphosphoric acid (50 g) and heated to 493 K under an N_2_ atmosphere for 4 h. The reaction mixture was cooled to room temperature and poured into 10% K_2_CO_3_(aq) solution. The precipitate was filtered under reduced pressure. Yellow crystals were obtained by recrystallization from methanol. Yield: 90%. Elemental analysis calculated: C 69.03, H 4.42, N 12.39 %; found: C 69.01, H 4.48, N 12.41 %.

### Refinement

All H atoms bound to C atoms were placed in geometrical positions with C—H = 0.93 Å and the *U*_iso_ values were constrained to be 1.2 times *U*_eq_ of the carrier atoms. Atoms H1A, H1B, H4A and H4B were located in difference Foruier maps and refined with N—H restrained to 0.86 (2) Å and with *U*_iso_(H) = 1.2*U*_eq_(N).

### Crystal data

**Table:**

C_13_H_10_N_2_S	*Z* = 4
*M**_r_* = 226.29	*F*(000) = 472
Triclinic, *P*1	*D*_x_ = 1.386 Mg m^−^^3^
Hall symbol: -P 1	Mo *K*α radiation, λ = 0.71073 Å
*a* = 8.7038 (5) Å	Cell parameters from 2375 reflections
*b* = 9.5933 (6) Å	θ = 2.3–26.3°
*c* = 14.5144 (9) Å	µ = 0.27 mm^−^^1^
α = 70.720 (1)°	*T* = 298 K
β = 77.326 (1)°	Block, blue
γ = 73.170 (1)°	0.30 × 0.20 × 0.20 mm
*V* = 1084.63 (11) Å^3^	

### Data collection

**Table:**

Bruker SMART CCD diffractometer	4199 independent reflections
Radiation source: fine-focus sealed tube	3123 reflections with *I* > 2σ(*I*)
graphite	*R*_int_ = 0.036
φ and ω scans	θ_max_ = 26.0°, θ_min_ = 1.5°
Absorption correction: multi-scan (SADABS; Sheldrick, 2003)	*h* = −10→10
*T*_min_ = 0.924, *T*_max_ = 0.948	*k* = −11→10
6984 measured reflections	*l* = −17→15

### Refinement

**Table:**

Refinement on *F*^2^	Primary atom site location: structure-invariant direct methods
Least-squares matrix: full	Secondary atom site location: difference Fourier map
*R*[*F*^2^ > 2σ(*F*^2^)] = 0.046	Hydrogen site location: inferred from neighbouring sites
*wR*(*F*^2^) = 0.115	H atoms treated by a mixture of independent and constrained refinement
*S* = 0.97	*w* = 1/[σ^2^(*F*_o_^2^) + (0.0596*P*)^2^] where *P* = (*F*_o_^2^ + 2*F*_c_^2^)/3
4199 reflections	(Δ/σ)_max_ = 0.001
301 parameters	Δρ_max_ = 0.29 e Å^−^^3^
4 restraints	Δρ_min_ = −0.23 e Å^−^^3^

### Special details

**Table:**

Geometry. All e.s.d.'s (except the e.s.d. in the dihedral angle between two l.s. planes) are estimated using the full covariance matrix. The cell e.s.d.'s are taken into account individually in the estimation of e.s.d.'s in distances, angles and torsion angles; correlations between e.s.d.'s in cell parameters are only used when they are defined by crystal symmetry. An approximate (isotropic) treatment of cell e.s.d.'s is used for estimating e.s.d.'s involving l.s. planes.
Refinement. Refinement of *F*^2^ against ALL reflections. The weighted *R*-factor *wR* and goodness of fit *S* are based on *F*^2^, conventional *R*-factors *R* are based on *F*, with *F* set to zero for negative *F*^2^. The threshold expression of *F*^2^ > σ(*F*^2^) is used only for calculating *R*-factors(gt) *etc*. and is not relevant to the choice of reflections for refinement. *R*-factors based on *F*^2^ are statistically about twice as large as those based on *F*, and *R*- factors based on ALL data will be even larger.

### Fractional atomic coordinates and isotropic or equivalent isotropic displacement parameters (Å^2^)

**Table:**

	*x*	*y*	*z*	*U*_iso_*/*U*_eq_	
C1	0.1360 (2)	0.6101 (2)	0.06950 (16)	0.0440 (5)	
C2	0.0112 (3)	0.7409 (3)	0.05957 (19)	0.0611 (7)	
H2	−0.0368	0.7833	0.0022	0.073*	
C3	−0.0402 (3)	0.8066 (3)	0.1348 (2)	0.0679 (7)	
H3	−0.1234	0.8943	0.1282	0.081*	
C4	0.0296 (3)	0.7447 (3)	0.2208 (2)	0.0634 (7)	
H4	−0.0078	0.7912	0.2711	0.076*	
C5	0.1539 (3)	0.6151 (3)	0.23330 (18)	0.0558 (6)	
H5	0.2007	0.5736	0.2911	0.067*	
C6	0.2066 (2)	0.5488 (2)	0.15651 (15)	0.0427 (5)	
C7	0.3187 (2)	0.4164 (2)	0.02866 (14)	0.0380 (5)	
C8	0.4141 (2)	0.3129 (2)	−0.02870 (14)	0.0372 (5)	
C9	0.3726 (2)	0.3255 (2)	−0.11916 (15)	0.0449 (5)	
H9	0.2833	0.4002	−0.1424	0.054*	
C10	0.4606 (3)	0.2302 (2)	−0.17460 (15)	0.0451 (5)	
H10	0.4295	0.2408	−0.2344	0.054*	
C11	0.5958 (2)	0.1178 (2)	−0.14273 (15)	0.0390 (5)	
C12	0.6382 (2)	0.1045 (2)	−0.05262 (16)	0.0452 (5)	
H12	0.7282	0.0304	−0.0297	0.054*	
C13	0.5487 (2)	0.1994 (2)	0.00275 (15)	0.0427 (5)	
H13	0.5788	0.1876	0.0630	0.051*	
C14	0.6126 (2)	0.2440 (2)	0.53255 (15)	0.0429 (5)	
C15	0.7512 (3)	0.2790 (3)	0.54271 (18)	0.0551 (6)	
H15	0.8010	0.2299	0.5992	0.066*	
C16	0.8130 (3)	0.3879 (3)	0.4673 (2)	0.0613 (7)	
H16	0.9060	0.4119	0.4731	0.074*	
C17	0.7399 (3)	0.4625 (3)	0.3830 (2)	0.0638 (7)	
H17	0.7843	0.5357	0.3332	0.077*	
C18	0.6027 (3)	0.4301 (3)	0.37176 (18)	0.0601 (7)	
H18	0.5535	0.4800	0.3151	0.072*	
C19	0.5396 (3)	0.3201 (2)	0.44794 (16)	0.0461 (5)	
C20	0.4087 (2)	0.1299 (2)	0.57195 (14)	0.0386 (5)	
C21	0.3035 (2)	0.0279 (2)	0.62886 (14)	0.0386 (5)	
C22	0.3356 (3)	−0.0669 (2)	0.72208 (15)	0.0467 (5)	
H22	0.4236	−0.0635	0.7472	0.056*	
C23	0.2405 (2)	−0.1649 (2)	0.77762 (16)	0.0483 (5)	
H23	0.2650	−0.2269	0.8396	0.058*	
C24	0.1078 (2)	−0.1729 (2)	0.74244 (16)	0.0440 (5)	
C25	0.0748 (3)	−0.0791 (2)	0.64972 (16)	0.0487 (5)	
H25	−0.0134	−0.0824	0.6248	0.058*	
C26	0.1713 (3)	0.0188 (2)	0.59423 (16)	0.0464 (5)	
H26	0.1472	0.0802	0.5321	0.056*	
N1	0.6820 (2)	0.0234 (2)	−0.19862 (15)	0.0554 (5)	
N2	0.20084 (19)	0.53292 (18)	−0.00148 (12)	0.0436 (4)	
N3	0.5371 (2)	0.13608 (18)	0.60184 (12)	0.0433 (4)	
N4	0.0104 (3)	−0.2687 (3)	0.80188 (16)	0.0600 (6)	
S1	0.35999 (7)	0.39025 (6)	0.14764 (4)	0.04624 (18)	
S2	0.36992 (7)	0.25484 (6)	0.45569 (4)	0.05063 (19)	
H1A	0.655 (3)	0.035 (3)	−0.2540 (13)	0.061*	
H1B	0.754 (2)	−0.048 (2)	−0.1747 (16)	0.061*	
H4A	−0.038 (3)	−0.292 (3)	0.7646 (15)	0.061*	
H4B	0.052 (3)	−0.339 (2)	0.8488 (15)	0.061*	

### Atomic displacement parameters (Å^2^)

**Table:**

	*U*^11^	*U*^22^	*U*^33^	*U*^12^	*U*^13^	*U*^23^
C1	0.0389 (12)	0.0461 (12)	0.0501 (14)	−0.0118 (10)	0.0011 (10)	−0.0207 (10)
C2	0.0518 (14)	0.0641 (15)	0.0686 (17)	0.0027 (12)	−0.0137 (12)	−0.0309 (13)
C3	0.0492 (14)	0.0746 (18)	0.086 (2)	0.0000 (13)	−0.0046 (14)	−0.0468 (16)
C4	0.0532 (15)	0.0781 (17)	0.0731 (18)	−0.0155 (13)	0.0060 (13)	−0.0494 (15)
C5	0.0573 (15)	0.0649 (15)	0.0536 (15)	−0.0189 (12)	−0.0015 (12)	−0.0279 (12)
C6	0.0395 (12)	0.0474 (12)	0.0452 (13)	−0.0169 (10)	0.0010 (10)	−0.0170 (10)
C7	0.0390 (11)	0.0404 (11)	0.0366 (11)	−0.0167 (9)	−0.0025 (9)	−0.0089 (9)
C8	0.0369 (11)	0.0370 (11)	0.0377 (11)	−0.0124 (9)	−0.0014 (9)	−0.0101 (9)
C9	0.0441 (12)	0.0447 (12)	0.0411 (12)	−0.0027 (10)	−0.0088 (10)	−0.0110 (10)
C10	0.0524 (13)	0.0480 (12)	0.0342 (12)	−0.0072 (10)	−0.0096 (10)	−0.0127 (10)
C11	0.0380 (11)	0.0385 (11)	0.0412 (12)	−0.0124 (9)	0.0004 (9)	−0.0128 (9)
C12	0.0408 (12)	0.0438 (11)	0.0485 (13)	−0.0029 (9)	−0.0113 (10)	−0.0130 (10)
C13	0.0450 (12)	0.0459 (12)	0.0395 (12)	−0.0110 (10)	−0.0099 (10)	−0.0123 (10)
C14	0.0477 (12)	0.0369 (11)	0.0430 (13)	−0.0072 (10)	−0.0016 (10)	−0.0154 (10)
C15	0.0570 (14)	0.0537 (14)	0.0592 (15)	−0.0143 (12)	−0.0062 (12)	−0.0222 (12)
C16	0.0586 (15)	0.0556 (14)	0.0763 (19)	−0.0214 (12)	0.0015 (14)	−0.0275 (14)
C17	0.0738 (18)	0.0478 (14)	0.0643 (17)	−0.0206 (13)	0.0050 (14)	−0.0128 (12)
C18	0.0688 (17)	0.0495 (14)	0.0535 (15)	−0.0147 (12)	−0.0028 (13)	−0.0066 (12)
C19	0.0495 (13)	0.0382 (11)	0.0469 (13)	−0.0054 (10)	−0.0010 (10)	−0.0152 (10)
C20	0.0432 (12)	0.0385 (11)	0.0330 (11)	−0.0027 (9)	−0.0038 (9)	−0.0155 (9)
C21	0.0401 (11)	0.0398 (11)	0.0358 (11)	−0.0049 (9)	−0.0019 (9)	−0.0167 (9)
C22	0.0422 (12)	0.0530 (13)	0.0462 (13)	−0.0136 (10)	−0.0106 (10)	−0.0108 (10)
C23	0.0478 (13)	0.0527 (13)	0.0420 (13)	−0.0140 (10)	−0.0092 (10)	−0.0064 (10)
C24	0.0391 (12)	0.0446 (12)	0.0490 (13)	−0.0067 (10)	−0.0006 (10)	−0.0203 (10)
C25	0.0426 (12)	0.0585 (14)	0.0534 (14)	−0.0124 (10)	−0.0099 (11)	−0.0243 (11)
C26	0.0494 (13)	0.0520 (13)	0.0379 (12)	−0.0059 (10)	−0.0100 (10)	−0.0156 (10)
N1	0.0568 (13)	0.0576 (12)	0.0497 (13)	0.0022 (10)	−0.0113 (10)	−0.0237 (11)
N2	0.0417 (10)	0.0446 (10)	0.0435 (10)	−0.0072 (8)	−0.0050 (8)	−0.0145 (8)
N3	0.0491 (11)	0.0456 (10)	0.0366 (10)	−0.0102 (8)	−0.0058 (8)	−0.0143 (8)
N4	0.0548 (13)	0.0675 (14)	0.0621 (15)	−0.0266 (11)	−0.0061 (11)	−0.0147 (11)
S1	0.0501 (3)	0.0474 (3)	0.0425 (3)	−0.0087 (3)	−0.0085 (3)	−0.0156 (2)
S2	0.0553 (4)	0.0491 (3)	0.0427 (3)	−0.0094 (3)	−0.0117 (3)	−0.0062 (3)

### Geometric parameters (Å, °)

**Table:**

C1—N2	1.391 (3)	C15—C16	1.375 (3)
C1—C2	1.391 (3)	C15—H15	0.930
C1—C6	1.399 (3)	C16—C17	1.384 (3)
C2—C3	1.367 (3)	C16—H16	0.930
C2—H2	0.930	C17—C18	1.372 (3)
C3—C4	1.383 (3)	C17—H17	0.930
C3—H3	0.930	C18—C19	1.392 (3)
C4—C5	1.380 (3)	C18—H18	0.930
C4—H4	0.930	C19—S2	1.732 (2)
C5—C6	1.390 (3)	C20—N3	1.307 (2)
C5—H5	0.930	C20—C21	1.460 (3)
C6—S1	1.730 (2)	C20—S2	1.7543 (19)
C7—N2	1.305 (2)	C21—C26	1.388 (3)
C7—C8	1.461 (3)	C21—C22	1.393 (3)
C7—S1	1.763 (2)	C22—C23	1.370 (3)
C8—C13	1.390 (3)	C22—H22	0.930
C8—C9	1.395 (3)	C23—C24	1.392 (3)
C9—C10	1.371 (3)	C23—H23	0.930
C9—H9	0.930	C24—N4	1.383 (3)
C10—C11	1.391 (3)	C24—C25	1.387 (3)
C10—H10	0.930	C25—C26	1.377 (3)
C11—N1	1.366 (3)	C25—H25	0.930
C11—C12	1.392 (3)	C26—H26	0.930
C12—C13	1.371 (3)	N1—H1B	0.819 (15)
C12—H12	0.930	N1—H1A	0.850 (15)
C13—H13	0.930	N4—H4A	0.869 (15)
C14—C19	1.388 (3)	N4—H4B	0.844 (15)
C14—N3	1.390 (3)	S1—S2	4.2373 (8)
C14—C15	1.391 (3)		
			
N2—C1—C2	125.4 (2)	C15—C16—H16	119.3
N2—C1—C6	115.50 (18)	C17—C16—H16	119.3
C2—C1—C6	119.1 (2)	C18—C17—C16	121.0 (2)
C3—C2—C1	119.5 (2)	C18—C17—H17	119.5
C3—C2—H2	120.3	C16—C17—H17	119.5
C1—C2—H2	120.3	C17—C18—C19	117.8 (2)
C2—C3—C4	121.0 (2)	C17—C18—H18	121.1
C2—C3—H3	119.5	C19—C18—H18	121.1
C4—C3—H3	119.5	C14—C19—C18	121.6 (2)
C5—C4—C3	121.3 (2)	C14—C19—S2	109.70 (16)
C5—C4—H4	119.4	C18—C19—S2	128.71 (19)
C3—C4—H4	119.4	N3—C20—C21	123.87 (18)
C4—C5—C6	117.7 (2)	N3—C20—S2	114.84 (15)
C4—C5—H5	121.1	C21—C20—S2	121.30 (15)
C6—C5—H5	121.1	C26—C21—C22	117.29 (19)
C5—C6—C1	121.5 (2)	C26—C21—C20	123.02 (18)
C5—C6—S1	129.30 (18)	C22—C21—C20	119.69 (18)
C1—C6—S1	109.24 (16)	C23—C22—C21	121.5 (2)
N2—C7—C8	124.89 (18)	C23—C22—H22	119.2
N2—C7—S1	114.76 (15)	C21—C22—H22	119.2
C8—C7—S1	120.34 (14)	C22—C23—C24	120.8 (2)
C13—C8—C9	117.06 (19)	C22—C23—H23	119.6
C13—C8—C7	122.61 (18)	C24—C23—H23	119.6
C9—C8—C7	120.33 (17)	N4—C24—C25	122.4 (2)
C10—C9—C8	121.48 (18)	N4—C24—C23	119.4 (2)
C10—C9—H9	119.3	C25—C24—C23	118.1 (2)
C8—C9—H9	119.3	C26—C25—C24	120.7 (2)
C9—C10—C11	120.95 (19)	C26—C25—H25	119.7
C9—C10—H10	119.5	C24—C25—H25	119.7
C11—C10—H10	119.5	C25—C26—C21	121.5 (2)
N1—C11—C10	120.28 (19)	C25—C26—H26	119.2
N1—C11—C12	121.75 (18)	C21—C26—H26	119.2
C10—C11—C12	117.97 (19)	C11—N1—H1B	117.7 (17)
C13—C12—C11	120.69 (19)	C11—N1—H1A	120.2 (16)
C13—C12—H12	119.7	H1B—N1—H1A	122 (2)
C11—C12—H12	119.7	C7—N2—C1	110.94 (17)
C12—C13—C8	121.86 (19)	C20—N3—C14	110.98 (17)
C12—C13—H13	119.1	C24—N4—H4A	108.7 (16)
C8—C13—H13	119.1	C24—N4—H4B	116.1 (17)
C19—C14—N3	115.20 (19)	H4A—N4—H4B	118 (2)
C19—C14—C15	119.69 (19)	C6—S1—C7	89.56 (10)
N3—C14—C15	125.1 (2)	C6—S1—S2	90.73 (7)
C16—C15—C14	118.5 (2)	C7—S1—S2	164.85 (6)
C16—C15—H15	120.8	C19—S2—C20	89.27 (10)
C14—C15—H15	120.8	C19—S2—S1	95.40 (7)
C15—C16—C17	121.4 (2)	C20—S2—S1	157.02 (7)
			
N2—C1—C2—C3	−179.3 (2)	S2—C20—C21—C22	−178.30 (15)
C6—C1—C2—C3	−0.2 (3)	C26—C21—C22—C23	−0.2 (3)
C1—C2—C3—C4	−0.2 (4)	C20—C21—C22—C23	−179.62 (19)
C2—C3—C4—C5	0.3 (4)	C21—C22—C23—C24	0.0 (3)
C3—C4—C5—C6	0.0 (4)	C22—C23—C24—N4	−177.5 (2)
C4—C5—C6—C1	−0.4 (3)	C22—C23—C24—C25	0.1 (3)
C4—C5—C6—S1	178.68 (17)	N4—C24—C25—C26	177.6 (2)
N2—C1—C6—C5	179.73 (18)	C23—C24—C25—C26	0.1 (3)
C2—C1—C6—C5	0.5 (3)	C24—C25—C26—C21	−0.4 (3)
N2—C1—C6—S1	0.5 (2)	C22—C21—C26—C25	0.4 (3)
C2—C1—C6—S1	−178.76 (17)	C20—C21—C26—C25	179.80 (19)
N2—C7—C8—C13	173.49 (18)	C8—C7—N2—C1	−179.01 (17)
S1—C7—C8—C13	−5.9 (3)	S1—C7—N2—C1	0.4 (2)
N2—C7—C8—C9	−6.2 (3)	C2—C1—N2—C7	178.6 (2)
S1—C7—C8—C9	174.43 (15)	C6—C1—N2—C7	−0.6 (3)
C13—C8—C9—C10	0.0 (3)	C21—C20—N3—C14	−179.77 (17)
C7—C8—C9—C10	179.73 (18)	S2—C20—N3—C14	0.5 (2)
C8—C9—C10—C11	−0.5 (3)	C19—C14—N3—C20	−0.5 (3)
C9—C10—C11—N1	179.81 (19)	C15—C14—N3—C20	179.33 (19)
C9—C10—C11—C12	0.4 (3)	C5—C6—S1—C7	−179.4 (2)
N1—C11—C12—C13	−179.2 (2)	C1—C6—S1—C7	−0.23 (15)
C10—C11—C12—C13	0.2 (3)	C5—C6—S1—S2	15.8 (2)
C11—C12—C13—C8	−0.7 (3)	C1—C6—S1—S2	−165.07 (14)
C9—C8—C13—C12	0.6 (3)	N2—C7—S1—C6	−0.08 (15)
C7—C8—C13—C12	−179.14 (18)	C8—C7—S1—C6	179.33 (16)
C19—C14—C15—C16	−0.7 (3)	N2—C7—S1—S2	91.1 (3)
N3—C14—C15—C16	179.4 (2)	C8—C7—S1—S2	−89.5 (3)
C14—C15—C16—C17	0.4 (3)	C14—C19—S2—C20	0.00 (15)
C15—C16—C17—C18	−0.1 (4)	C18—C19—S2—C20	179.6 (2)
C16—C17—C18—C19	0.1 (4)	C14—C19—S2—S1	−157.47 (14)
N3—C14—C19—C18	−179.35 (19)	C18—C19—S2—S1	22.1 (2)
C15—C14—C19—C18	0.8 (3)	N3—C20—S2—C19	−0.31 (16)
N3—C14—C19—S2	0.3 (2)	C21—C20—S2—C19	179.97 (17)
C15—C14—C19—S2	−179.57 (16)	N3—C20—S2—S1	101.9 (2)
C17—C18—C19—C14	−0.5 (3)	C21—C20—S2—S1	−77.8 (2)
C17—C18—C19—S2	179.95 (18)	C6—S1—S2—C19	−101.16 (9)
N3—C20—C21—C26	−177.40 (18)	C7—S1—S2—C19	167.8 (3)
S2—C20—C21—C26	2.3 (3)	C6—S1—S2—C20	157.87 (19)
N3—C20—C21—C22	2.0 (3)	C7—S1—S2—C20	66.9 (3)

### Hydrogen-bond geometry (Å, °)

**Table:**

*D*—H···*A*	*D*—H	H···*A*	*D*···*A*	*D*—H···*A*
N1—H1B···N4^i^	0.82 (2)	2.64 (2)	3.374 (3)	149 (2)
N1—H1A···N3^ii^	0.85 (2)	2.32 (2)	3.145 (3)	163 (2)

Symmetry codes: (i) *x*+1, *y*, *z*−1; (ii) *x*, *y*, *z*−1.
